# Antibody-Based Immunotherapeutic Strategies for the Treatment of Hematological Malignancies

**DOI:** 10.1155/2020/4956946

**Published:** 2020-09-17

**Authors:** Yizhao Han, Zhuojun Liu, Jia Liu, Weiqi Yan, Yuanshi Xia, Shuhua Yue, Jian Yu

**Affiliations:** ^1^Beijing Advanced Innovation Center for Biomedical Engineering, Beihang University, Beijing 100083, China; ^2^School of Biological Science and Medical Engineering, Beihang University, Beijing 100083, China

## Abstract

As the most common type of cancer in the world, hematological malignancies (HM) account for 10% of all annual cancer deaths and have attracted more attention. Conventional treatments, such as chemotherapy, radiotherapy, and hematopoietic stem cell transplantation (HSCT), could relieve patients suffering HM. However, serious side effects and high costs bring patients both physical complaints and mental pressure. Recently, compared with conventional therapeutic strategies for HM patients, antibody-based immunotherapies, including cancer vaccines, oncolytic virus therapies, monoclonal antibody treatments, and CAR-T cell therapies, have displayed longer survival time and fewer adverse reactions, even though specific efficacy and safety of these antibody-based immunotherapies still need to be evaluated and improved. This review summarized the advantages of antibody-based immunotherapies over conventional treatments, as well as its existing difficulties and solutions, thereby enhancing the understanding and applications of antibody-based immunotherapies in HM treatment.

## 1. Introduction

The American Cancer Society released the American cancer survival statistics report for 2019 on A Cancer Journal for Clinicians and predicted that 1,762,450 cancer cases would be diagnosed in the United States in 2019. Hematological malignancies (HM), including lymphoma, myeloma, and leukemia, would account for approximately 10% of the new cases, and 9.35% of the estimated deaths [1]. Conventionally, the first-line HM treatments are based on chemotherapy, radiotherapy, and hematopoietic stem cell transplantation (HSCT) [2]. According to the National Cancer Institute's (NCI's) Surveillance, Epidemiology, and End Results (SEER) Program, under the conventional therapies, the 5-year survival rate of leukemia, Hodgkin's lymphoma (HL), and non-Hodgkin's lymphoma (NHL) is 60.3%, 87.7%, and 71.4%, respectively, in the United States [3]. Although the first-line treatments are efficient, they have a high risk of relapse. As two nonspecific methods, chemotherapy and radiotherapy damage healthy cells and are not sufficient to eliminate the diseases. HSCT is a more effective therapy for leukemia and lymphoma, but the high level of graft versus host disease (GVHD) might lead to the death of patients. Except for these conventional treatments, numerous patients with HM have obtained benefits from tumor antibody-based immunotherapies over the past 10 decades, and experts are continuing to explore novel antibody-based immunotherapeutic strategies. This review compared the advantages and disadvantages of the conventional therapies with antibody-based immunotherapies for the treatment of HM and summarized the existing antibody-based immunotherapies.

## 2. Antibody-Based Immunotherapies versus Conventional Therapies for the Treatment of HM

The conventional treatment methods for HM include chemotherapy, radiotherapy, and HSCT. Chemotherapy is effective in overwhelming cancer outgrowth and prolonging patients' life, whereas several factors such as cancer staging and the chemotherapy tolerance of patients always determine different therapeutic effectiveness for different patients. Furthermore, the toxicity of chemotherapeutic drugs affects tumor cells and the normal cells in parallel, which results in the damage of the immune system and causes a variety of physical disorders in patients such as nausea, vomiting, hair loss, cognitive disorder, and blood cell reduction. Compared with chemotherapy, radiotherapy focuses on the surrounding tissues of the tumor, while the normal tissues around the field of radiotherapy are extremely harmed by direct radiation exposure during radiotherapy, which may lead to the hemogram decline, immune dysfunction, skin erosion, and tissue inflammation and necrosis as well. In recent years, even if some patients would relapse after transplantation, HSCT has become a mainstream strategy for the treatment of HM, because it can provide a longer survival period and better quality of life compared with chemotherapy and radiotherapy. The main classifications of HSCT include autologous HSCT (AHSCT) and allogeneic HSCT (allo-HSCT). AHSCT avoids graft rejection and GVHD and involves few complications. However, due to the lack of graft-versus-tumor (GVT) and the possibility that the graft may mix tumor cells, AHSCT may have a high relapse rate. The allo-HSCT cells are collected from normal donors without tumor cell contamination and have an antitumor immunological effect due to the low relapse rate and high disease-free survival (DFS). However, under allo-HSCT treatment, patients should take immunosuppressants for long periods of time to avoid immunologic rejection, which may cause diverse infections, such as bacteria, viruses, fungi, and protozoan [4].

In recent years, due to its superiority in therapeutic efficacy, side effects, and prices than conventional therapies, increasing attention has been paid to antibody-based immunotherapies. Antibody-based immunotherapies, including cancer vaccines, oncolytic virus therapies, chimeric antigen receptor T cells (CAR-T cells), and monoclonal antibodies, can train the immune system to attack cancer cells and have been introduced in the following sections [5].

## 3. The Principle and the Application of Antibody-Based Immunotherapies in HM

### 3.1. Cancer Vaccines

Tumor nonspecific and specific vaccines with high safety and rare adverse immune reactions have become an attractive strategy to treat HM. The most common adverse immune reactions of cancer vaccines include fever or painful swelling of the injected lymph node (flu-like symptoms) [6]. Cancer vaccines can induce antitumor immune responses through recognizing tumor-associated antigens (TAAs); however, the clinical application of cancer vaccines is limited due to the low immunogenicity of most TAAs [7]. Since one or more tumor-associated antigen peptide vaccines cannot control the development of tumors, better therapeutic effects are obtained by using intact tumors or their derivative lysates as vaccines. Firstly, whole tumor cells can elicit broad-spectrum immune responses to different TAAs. Secondly, they promote the cross-presentation of antigens to CD4^+^ and CD8^+^ T cells, which result in the long-term antitumor memory of CD4^+^ and CD8^+^ T cells after tumor fragments are internalized by antigen-presenting cells (APCs) [8].

**Figure 1:**
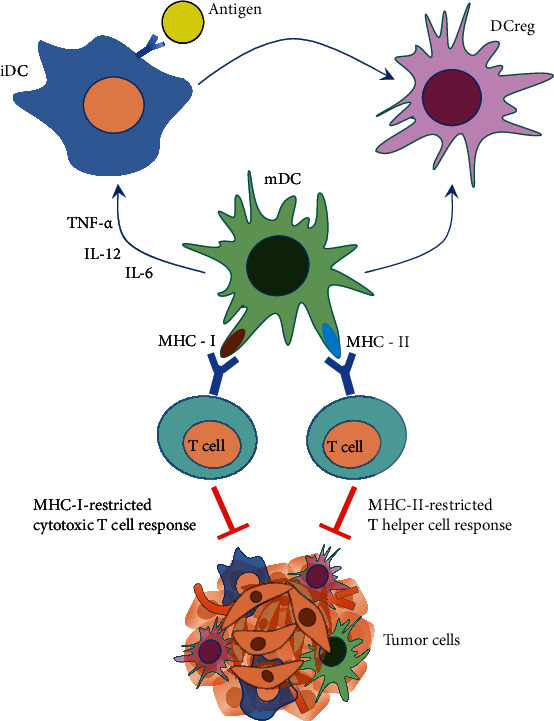


Previous studies indicate that dendritic cells (DCs) are the most effective specialized APCs in the human's immune system [9], because DCs cannot only induce the formation of specific cytotoxic T lymphocytes (CTL) but also acquire and process antigens, subsequently, presenting them to lymphoid tissues and controlling specific immunity activation. There are three forms of DCs: steady-state immature DCs (iDCs), mature DCs (mDCs), and regulatory DCs (DCregs). In the process of antigen presentation, iDCs firstly capture and devour antigens and then secrete tumor necrosis factor-*α* (TNF-*α*), interleukin-12 (IL-12), and interleukin-6 (IL-6), which promote the maturation of iDCs to mDCs. With a high expression of major histocompatibility complex (MHC)-I/II, costimulatory molecules (ICOS), and adhesion molecule (ICAM), mDCs have powerful antigen-presenting ability, and the antigen is presented to T cell in the T cell area of the lymph nodes, to start the MHC-I-restricted cytotoxic T cell response and MHC-II-restricted T helper cell response (Figure 1).

The patients suffering from multiple myeloma (MM) can be successfully cured by the DC-loaded (DC-LD) autologous immunoglobulin cancer vaccines, and after loading, the immunogenic foreign carrier protein keyhole limpet hemocyanin (KLH) cancer vaccines can be recognized by T cells. After high-dose chemotherapy and AHSCT, 12 MM patients who were intravenously injected with DC-ID and Id-KLH (the unique idiotype of follicular NHL expression, Id) have produced Id-specific CTL immunity with mild and temporary side effects; in addition, their DFS was significantly longer than the control group, suggesting the efficacy of the combination therapy of DC vaccine with chemotherapy or AHSCT in MM treatment [10]. Moreover, some scholars reported that heat shock protein GP96 [11] and vascular endothelial growth factor-1 (VEGF-1) [12] could also be applied as target antigens in HM.

However, HM can induce a large number of immunosuppressive cells in patients, which may affect the maturation and function of DCs. For example, chronic lymphocytic leukemia (CLL) can induce the production of IDO^hi^CD14^+^HLA-DR^lo^ myeloid-derived suppressor cells (MDSCs), a kind of heterogeneous and immature myeloid progenitor cells, and have an immunosuppressive function. Obtained from healthy donors, monocytes can be transformed into IDO^hi^ MDSCs in vitro by CLL cells, which may lead to the relapse of CLL [13, 14].

### 3.2. Oncolytic Virus (OV) Therapies

As a class of natural or genetically modified viruses, OV plays an antitumor role by selectively destroying tumor cells and eliciting the body's specific cellular immune response. Although the cytotoxicity on tumor cells of OV has not been entirely clarified, the possibility depends on the theory about the mass replication of the virus and inducing apoptosis in tumor cells [15, 16]. Extensive researches about OV focus on measles virus (MV), reovirus (RV), coxsackievirus (CV), and vesicular stomatitis virus (VSV) for the treatment of HM [17].

MV is a kind of negative-sense, single-strand RNA virus, which is classified as Paramyxoviridae morbillivirus. The wild-type MV exhibits a natural tropism for lymphocytes, macrophages, and dendritic cells. In the 1970s, a great deal of evidence substantiated that patients suffering from leukemia, Burkitt lymphoma, and HL had relieved after MV infection. Therefore, MV was identified as a potential therapeutic strategy for HM at that time [18, 19]. Peng et al. demonstrated that measles viruses of the Edmonston lineage (MV-Edm) strain could extensively replicate in a variety of MM samples isolated from 6 patients *in vitro*, and *in vivo*, intratumoral or intravenous MV injection could also significantly lessen the tumoral volume in tumor-bearing mice [20]. Most importantly, the MV-sodium iodide symporter (NIS) oncolytic virus has the potential to provide targeted radiotherapy to tumor sites. NIS is a glycoprotein expressed in thyroid follicular cells (TFC); through NIS, iodine can be transported into the tumor cells against the concentration gradient, which is the basis for the radioactive iodine treatment of differentiated thyroid cancer. In MV-resistant MM tumor models, combined with radiotherapy, MV-NIS enhances the antitumor effect significantly [21]. However, MV has several side effects such as pneumonia, laryngotracheobronchitis, encephalitis, and occasionally leading to death. RV belongs to the nonenveloped double-stranded RNA virus of Reoviridae. Alain et al. discovered that MM cell lines were susceptible to RV. An animal experiment proved that RV could effectively inhibit the growth of Raji cells (Burkitt's lymphoma cell line) in mice [22]. What is more, RV can directly kill acute myeloblastic leukemia (AML) cells [23]. When combined with targeted drugs such as rituximab, the cytotoxicity of RV in CLL cells can be increased significantly [24]. RV treatments of patients HM might lead to viremia [25]. VSV, a negative-sense, single-strand RNA virus, usually infects cattle, horses, and pigs but rarely infects humans. The symptoms of human infection are usually asymptomatic or influenza-like symptoms. Due to the limited prevalence of VSV in humans, most patients do not have natural antibodies, making VSV be an ideal OV. An ongoing phase I clinical trial (NCT03017820) is aimed at evaluating the tolerance and safety of vesicular stomatitis virus-interferon-*β*-sodium iodide symporter (VSV-IFN-*β*-NIS) in the treatment of MM, AML, or T cell lymphoma, which showed neurotoxicity through this trial [26]. As a subtype of nonenveloped positive-strand RNA CV viruses, CVA21 (coxsackievirus A21) has the toxicity to MM cell lines and CD138^+^ MM cells isolated from patients [27]. However, CVA21 might cause myositis, which may hinder its clinical effect. To solve this problem, muscle-specific miRNAs were inserted into the 3′ nontranslational region of the CVA21 genome. After the intratumoral injection of these CVA21, mice had a long survival time without myositis side effect, while the wild-type CVA21 mice developed significant muscle inflammation and necrosis [28]. In addition, no serious side effects of CV were found in clinical trials [29].

In order to improve the curative effect of OV, cell carrier and combination therapy were conducted. Several clinical studies have found that even if there are efficiently neutralizing antibodies in patients, a small number of OV viruses can still reach the tumor site and play the oncolytic role effectively [30]. It has become an effective strategy that OV is adsorbed on cells with tumor recognition ability, which helps the viruses cross the complex blood environment to the tumor site by using the tendency of the cell toward tumor tissue. Known cell carriers include tumor-specific cytotoxic T lymphocyte, DC, mesenchymal stem cells (MSCs), and CAR-T [31–33]. The combination treatment of OV and cell carriers with chemotherapy or HSCT is effective for the treatment of HM. For example, as a recognized drug for the treatment of MM, cyclophosphamide can improve the efficacy of OV by downregulating organism antiviral immune response with cell carriers [34].

### 3.3. Monoclonal Antibodies (mAbs)

mAbs are derived from the cloning of a single B lymphocyte or a hybridoma and only recognize specific epitopes with the main characteristics including high uniformity, single bioactivity, and high specificity. Several mAbs have been approved by the FDA for the treatment of HM. Rituximab, a chimeric anti-CD20 mAb, is one of the biological agents with a wide range of applications, which revolutionizes the treatment of B cell HM. Rituximab has been used to treat relapsed NHL and CLL since 1997. In 2006, the combination of rituximab with cyclophosphamide, vincristine, and prednisone (CVP) was approved by the FDA for the treatment of low-grade NHL and CD20^+^ follicular lymphoma, which the side effects occurred, including fatigue, neutropenia, and back pain [35]. Ofatumumab is a humanized type I anti-CD20 mAb, which is approved by the FDA for the treatment of naïve and relapsed/refractory (R/R) CLL patients. A single-arm study assessed the effect of ofatumumab in the refractory CLL patients who had inconspicuous therapeutic effects of fludarabine and alemtuzumab, and almost half of patients improved their response rate and complete response (CR); however, the side effects also occurred, including infections, cough, diarrhea, anemia, fatigue, fever, neutropenia, dyspnea, nausea, and rash [36]. In another clinical trial, the combination of ofatumumab with chemotherapy for the naïve CLL patients could significantly improve median progression-free survival (MPFS) and the median response time, without achieving overall survival (OS) for more than 28.9 months [37]. CD30, a member of the TNF receptor (TNFR) family, is a transmembrane glycoprotein and is highly expressed in HL and systemic anaplastic large cell lymphoma (sALCL) cells [38]. Brentuximab Vedotin (BV) is an antibody-drug conjugate (ADC), which contains anti-CD30 mAb covalently binding to antimicrotubule monomethyl auristatin E (MMAE) by a protease-cleavable linker. The combination of BV and CD30 leads to the internalization of the MMAE-CD30 compound and the release of MMAE by proteolytic and cleavage. In 2012, the FDA approved BV for the treatment of relapsed HL or relapsed sALCL [39]. However, BV may cause many adverse events, including peripheral sensory neuropathy, neutropenia, fatigue, nausea, diarrhea, fever, upper respiratory infection, and vomiting. Daratumumab, a humanized anti-CD38 mAb, was adopted with lenalidomide and dexamethasone or bortezomib and dexamethasone for the treatment of MM. However, drug resistance occurred in many patients who accepted daratumumab, which was connected with the increased expression levels of complementary inhibitory protein (CIPs), membrane cofactor protein (MCP) CD46, decay-accelerating factor CD55, and protective protein (CD59) [40]. Also, daratumumab treatment induced side effects, such as nasal congestion, cough, allergic rhinitis, throat irritation, and dyspnea [41]. The first immunotoxin, denileukin diftitox, was approved by the FDA to treat the relapsed cutaneous T cell lymphoma (CTCL). This fusion protein toxin functions by cutting off the combination between DT (DAB389) and IL-2 protein, for targeting CD25 subunits of IL-2 receptors [42]; however, it is always accompanied with asthenia, fever, nausea, mild hypotension, flu-like symptoms, myalgias, chills, and vomiting [43].

The therapies focusing on immune checkpoints and their ligands indicate impressive response and persistent response in HM. Immune checkpoints are a class of immunosuppressive molecules, which can avoid damage and destruction of normal tissues by regulating the intensity and breadth of the immune response. In recent years, it has been found that tumor cells can escape from the surveillance of the immune system by activating immune checkpoints. Immune checkpoint inhibitors can promote T cell activation and produce antitumor immune effects. For HM, the main immune checkpoints include CTLA-4 [44], PD-1, LAG-3 [45], and TIM-3 [46]. In 2016, a PD-1 inhibitor nivolumab was approved by the FDA as a treatment for R/R HL patients who relapsed or deteriorate after ASCT, which might be accompanied with fatigue, infusion reaction, arthralgia, and rash [47]. In the Ib-013 study, 31 R/R HL patients who had accepted BV were treated with pembrolizumab, another PD-1 inhibitor, and had a 65% objective response rate (ORR) at 12 weeks, and 90% of patients manifested tumor shrinkage during treatment, but from which, several side effects such as pneumonitis and nephrotic syndrome occurred in certain patients [48].

### 3.4. CAR-T Cell Therapies

Due to the tumor-antigen-specific binding, CAR-T cell therapy has exhibited significant remission rates for the treatment of HM, such as MM, acute lymphocytic leukemia (ALL), and CLL. The CAR molecules consist of antigen-recognizing single-chain fragment variable (scFv) and T cell-activating domain (including the CD3 zeta chain), which are integrated into T cells by gene engineering (such as retrovirus or lentivirus). CAR is designed to identify specific antigens, which can elicit CAR-T activation without the traditional restriction from specific T cell receptor and MHC [49, 50].

Two CAR-T19 cell products were approved for the treatment of B-ALL and diffuse large B cell lymphoma (DLBCL) by the FDA: tisagenlecleucel with 4-1BB ICOS and axicabtagene ciloleucel with CD28 ICOS. The application of CAR-T cell had altered the therapy of R/R B-ALL, which increased the complete response rate (CRR) from 29% to more than 80% in adults [51–53]. In particular, a single-phase I-IIa tis-cel clinical research carried out by Children's Hospital of Philadelphia and the University of Pennsylvania illustrated a CRR of 93% involving 60 children and young people with R/R B-ALL [54]. The phase II clinical trial results from the University of Pennsylvania, NCI, and Memorial Sloan-Kettering Cancer Center revealed that about 40% of the B-NHL patients could get continuous completely CR by CAR-T19 therapy [55–57]. Although CAR-T cell therapy is effective in B-ALL, it has the risk of relapse and some adverse reactions, such as B cell lacking, cytokine release syndrome (CRS) [58], and varied degrees of neurotoxicity [59]. Studies show the low dose level of CAR-T cell (<1 × 10^5^ kg^−1^) is a possible reason for the rapid relapse after CAR-T treatment. For patients with a low dose level of CAR-T cell, after the combination treatment with HSCT, the median OS was significantly better than the patients without HSCT [60]. Follow-up treatment with HSCT did not improve the survival of patients with deep minimal residual disease (MRD) negative remission. However, HSCT may also fail to eliminate the growth of CD19-negative ALL tumor cells after CAR-T treatment [61], so it is debatable on the significance of CAR-T treatment bridging HSCT.

An ideal target for the treatment of CAR-T for MM is B cell mature antigen (BCMA, also known as TNFRSF17), since its overexpression on malignant plasma cells [62, 63]. The University of Pennsylvania reported that combined with high-dose cyclophosphamide, CAR-T-BCMA with 4-1BB ICOS demonstrated a high clinical reaction rate (64%), and the incidence of CRS and neurotoxicity were 32% and 12%, respectively [64]. As an agent targeting BCMA, L CAR-B38M was targeted at CAR-T cells with 41BB costimulating molecules. The latest clinical trial results for the treatment of R/R MM indicated that the ORR was 88.2%, the stringent CRR was 76%, and the incidence above 3 grades of CRS was 35%, which showed good efficacy. According to the clinical trials of CAR T-BCMA therapy, CRS was the common adverse effect and its degree was possibly associated with the BCMA abundance on the surface of myeloma cells, BCMA-positive tumor burden, and cytokine IL-6 profiles [65]. Except for BCMA, other targets of CAR-T cells for the treatment for MM, such as NY-Eso1, kappa light chain, CD44 subtypes variant 6 (CD44v6), CD56, CD38, and CD138, have been applied to basic researches and clinical trials. However, there are few clinical MM cases with the above antitarget; thus, their clinical therapeutic effect cannot be determined yet [66].

Although CLL is a common B cell chronic lymphocytic proliferative disease with a 5-year survival rate of 79.2%, it is still an incurable disease, especially R/R CLL with a very poor prognosis [67]. From 2011, CAR-T19 has been used in the treatment of CLL with a response rate of 50%-70% and the CRR of 20%-30% that are significantly lower than those in the ALL and B-NHL treatment. The poor CAR-T therapeutic effect in CLL may be caused by the abnormal upregulation of depletion indicators (including PD1, CD244, and CD160) on T cell surface, the lack of memory cells, and the low proliferation and cytotoxicity of CD8-positive T cells in CLL patients [68, 69]. The combination of CAR-T with ibrutinib is ongoing to improve the therapeutic efficacy for CLL treatment [70].

Compared with B cell malignancies, the application of CAR-T in T cell malignancies is more challenging. There are two difficulties in T cell malignancy treatment: the acquirement of T cell-specific antigens and the serious cannibalism between T cells in the CAR-T preparation [71]. In order to solve this problem, an effective method is to knock out the targeted antigens on the T cell surface by CRISPR/Cas9 technology [72]. Most importantly, the use of NK cells or *γδ*T cells to prepare CAR molecules and the application of transient gene modification technology or suicide gene installation can avoid the mutual killing of T cells [73].

## 4. Challenges of Antibody-Based Immunotherapies

Antibody-based immunotherapy is evolving rapidly, which changes the mode of prognosis and treatment in HM. Due to the better understanding of antibody-based immunotherapy, more hypotheses are proposed and the relative studies are further performed. But antibody-based immunotherapy has its limitations that it is only curative for a subset of cancer types and patients in HM and has several side effects (Table 1). The main difficulties of cancer vaccines are the accurate selection of antigens, tumor antigen peptides, tumor lysates, or apoptotic tumor cells, which limit the therapeutic effects of vaccines. OV may induce a strong immune response for its strong immunogenicity, which decreases its inhibitory effect on tumor cells. As a major breakthrough in immunotherapy, CAR-T cell therapy is still limited by its side effects, including CRS, neurotoxicity, and B cell lacking. In addition, antigen loss might limit the efficiency of CAR-T cell therapy. For instance, hemizygous deletion and the frameshift and missense mutations of CD19 exon 2 were observed in 10%-20% pediatric B-ALL patients [74].

## 5. Conclusion

Compared with conventional radiotherapy, chemotherapy, and HSCT, antibody-based immunotherapies demonstrate a better therapeutic efficiency in the treatment of HM patients, which are shown by various preclinical and clinical studies. However, it is necessary to gain a deeper insight into the rational design of clinical trials of existing therapies and adopt the combinations of treatments to increase the efficiency and reduce the side effects of antibody-based immunotherapies as well.

## Figures and Tables

**Table 1:** Clinical applications and side effects of antibody-based immunotherapeutic strategies.

Strategies	Types	Clinical applications	Side effects
Cancer vaccines	DCs	MM, CLL	More frequently systemic flu-like symptoms occurred: fever or painful swelling of the injected lymph node

OV therapies	MV	Leukemia, Burkitt lymphoma, HL, MM	Pneumonia, laryngotracheobronchitis, encephalitis, occasionally leading to death
	RV	MM, Burkitt lymphoma, AML, CLL	Viremia
	VSV	MM, AML, T cell lymphoma	Neurotoxicity
	CV	MM	No serious side effects

mAbs	Rituximab	Low-grade NHL, CD20^+^ follicular lymphoma, CLL	Fatigue, neutropenia, back pain
	Ofatumumab	R/R CLL	Infections, cough, diarrhea, anemia, fatigue, fever, neutropenia, dyspnea, nausea, rash
	BV	HL, sALCL	Peripheral sensory neuropathy, neutropenia, fatigue, nausea, diarrhea, fever, upper respiratory infection, vomiting
	Daratumumab	MM	Nasal congestion, cough, allergic rhinitis, throat irritation, dyspnea
	Denileukin diftitox	CTCL	Asthenia, fever, nausea, mild hypotension, flu-like symptoms, myalgias, chills, vomiting
	Nivolumab	HL	Fatigue, infusion reaction, arthralgia, rash
	Pembrolizumab	HL	Pneumonitis, nephrotic syndrome

CAR-T cell therapies	CAR-T19	ALL, DLBCL, B-NHL, CLL	CRS, neurotoxicity, B cell lacking
	CAR-T-BCMA	MM	CRS, neurotoxicity
